# Impact of sarcopenia and frailty on outcomes of patients with sepsis or septic shock: a systematic review and meta-analysis

**DOI:** 10.3389/fnut.2025.1679632

**Published:** 2025-10-13

**Authors:** Fangchun Ni, Lingli Zheng

**Affiliations:** Department of Intensive Care Medicine, Xianlin Branch, Tongde Hospital of Zhejiang Province, Hangzhou, Zhejiang, China

**Keywords:** frailty, meta-analysis, sarcopenia, sepsis, systematic review

## Abstract

**Background:**

Sarcopenia and frailty are emerging risk factors that may modify outcomes in patients with sepsis or septic shock. This review aims to assess the association of sarcopenia and frailty with mortality, length of stay in the hospital and intensive care unit (ICU), and duration of mechanical ventilation in adults with sepsis.

**Methods:**

The PubMed, EMBASE, Scopus, CINAHL, Web of Science, and CENTRAL databases were searched from inception to May 31, 2025, for observational studies reporting outcomes stratified by sarcopenia and/or frailty in sepsis. Random-effects meta-analyses (Der Simonian–Laird) were done, and the data were presented as pooled odds ratios (OR) for mortality and weighted mean differences (WMD) for continuous outcomes. Cochran’s Q and *I*^2^ statistics quantified heterogeneity.

**Results:**

Thirty studies (*n* ≈ 38,000) were included. Sarcopenia (21 cohorts) was associated with higher in-hospital mortality (OR = 1.54; 95% CI: 1.03–2.30; *p* = 0.034; *I*^2^ = 85.5%), longer hospital (WMD = +5.37 days; 95% CI: 2.01–8.73; *p* = 0.002; *I*^2^ = 97.5%), and ICU (WMD = +1.49 days; 95% CI: 0.64–2.34; *p* = 0.001; *I*^2^ = 94.9%) stay and higher duration of mechanical ventilation (WMD = +0.99 days; 95% CI: −0.003 to 1.99; *p* = 0.051; *I*^2^ = 0%). Frailty (9 cohorts) demonstrated a non-significant trend toward increased mortality (OR = 1.68; 95% CI: 0.92–3.05; *p* = 0.091; *I*^2^ = 98.1%) but was linked to more extended ICU stay (WMD = +0.99 days; 95% CI: 0.69–1.28; *p* < 0.001; *I*^2^ = 78.1%). Frailty did not significantly affect hospital length of stay (WMD = –0.44 days; 95% CI: −5.71 to 4.84; *p* = 0.87; *I*^2^ = 99.8%).

**Conclusion:**

Sarcopenia independently predicts worse mortality and prolonged hospitalization in sepsis, underscoring the need for early muscle-preserving interventions. Frailty prolongs ICU stay and may inform shared-decision discussions, although its impact on mortality is less consistent.

**Systematic review registration:**

https://www.crd.york.ac.uk/PROSPERO/view/CRD420251058423, CRD420251058423.

## Introduction

Sepsis and septic shock are among the leading causes of morbidity and mortality worldwide, accounting for substantial intensive care unit (ICU) admissions, prolonged hospitalization, and elevated healthcare costs (1). Despite advances in early recognition and management, such as prompt antibiotic administration, hemodynamic optimization, and organ support, sepsis mortality rates can exceed 25–30%, particularly when complicated by septic shock (2). Outcomes vary widely based on patient factors such as age, comorbidities, and baseline physiological reserve, impacted by sarcopenia and frailty (3, 4). Sarcopenia refers to the loss of skeletal muscle mass and strength, while frailty represents a broader state of multisystem decline, often manifested by reduced physiological resilience to stressors (5). Growing evidence suggests that these conditions not only predispose individuals to adverse health events but also exacerbate the course and outcomes of acute critical illnesses such as sepsis (6).

Sarcopenia is recognized in geriatric medicine as a key determinant of functional independence, risk of falls, and overall quality of life (7). While its prevalence increases with age, with the incidence of up to 50% in individuals over 80 years, it also occurs in younger patients with chronic diseases, malignancies, or prolonged immobilization (8). The pathophysiology of sarcopenia involves a complex interplay between hormonal changes (e.g., reduced growth hormone and testosterone), chronic low-grade inflammation, mitochondrial dysfunction, and alterations in protein synthesis and degradation (9). In sepsis, these mechanisms are further amplified by systemic inflammation, metabolic derangements, and catabolic stress that may accelerate muscle wasting (10). Sepsis-induced muscle atrophy not only impairs ventilatory mechanics and prolongs mechanical ventilation but also compromises mobility and rehabilitation potential, which in turn can increase the length of ICU and hospital stay (11).

Frailty, although often studied in older adults, encompasses multiple domains of physiological decline, including weight loss, exhaustion, slowed gait speed, weakened grip strength, and low physical activity, which collectively lead to dysregulated immune function, impaired wound healing, and blunted stress responses (3). In septic patients, frailty has been linked to higher rates of organ dysfunction, increased need for vasopressors, and decreased likelihood of returning to baseline functional status (12). The cumulative deficits model of frailty postulates that each additional physiological impairment exponentially multiplies vulnerability (13). Therefore, frail septic patients are less able to compensate for the hemodynamic instability and metabolic demands that accompany the condition (13).

Multiple observational studies have evaluated the relationship between pre-existing sarcopenia or frailty and sepsis outcomes (14). In surgical and medical ICUs, patients with sarcopenia, often identified through computed tomography–derived measures of muscle cross-sectional area at L3 vertebral level, exhibit significantly higher in-hospital and 30-day mortality (15). These associations persist even after adjusting for age, severity of illness scores, and other comorbidities, suggesting that muscle mass serves as an independent prognostic marker (16). Similarly, frailty, often assessed using validated tools such as the Clinical Frailty Scale or Frailty Phenotype, has been associated with increased mortality, prolonged ICU length of stay, and elevated risk of discharge to long-term care facilities among septic patients (17). Furthermore, frailty appears to predict not only short-term survival but also long-term outcomes such as 90-day and 1-year mortality, as well as post-sepsis functional dependency (18).

Beyond mortality, both sarcopenia and frailty have been linked to prolonged duration of mechanical ventilation, a critical driver of ICU length of stay and nosocomial complications such as ventilator-associated pneumonia (19). Reduced respiratory muscle mass and strength in sarcopenic patients impair weaning success, often necessitating longer support and increasing the risk of ventilator-associated events (20). Frail patients, with their baseline reduced physiological reserve, may fail standardized spontaneous breathing trials and require prolonged ventilatory support, resulting in longer ICU stays (21). However, most existing studies on the association between sarcopenia, frailty, and sepsis outcomes are single-center, retrospective analyses with heterogeneous definitions of sarcopenia or frailty, making it difficult to generalize findings (22, 23).

The objective of this review was to assess the impact of sarcopenia and frailty with mortality, length of stay in the hospital and ICU, and duration of mechanical ventilation among adult patients with sepsis.

## Methods

This systematic review and meta-analysis were conducted and reported following the Preferred Reporting Items for Systematic Reviews and Meta-analyses (PRISMA) 2020 statement. Study protocol, eligibility criteria, information sources, search strategy, study selection, data collection, risk of bias assessment, and data synthesis were preregistered in PROSPERO (Registration ID: CRD420251058423).

### Eligibility criteria

#### Inclusion criteria

The inclusion criteria were as follows: (1) Original peer-reviewed studies (randomized trials, non-randomized trials, prospective or retrospective cohort studies, case–control studies, and cross-sectional analytical studies) that evaluated the association between either sarcopenia or frailty and clinical outcomes in adults (≥ 18 years) diagnosed with sepsis or septic shock; (2) Studies using validated measurement of sarcopenia (e.g., computed tomography [CT]–derived skeletal muscle index at L3) or frailty (e.g., Clinical Frailty Scale, Frailty Phenotype, or Frailty Index) and assessed at baseline (pre-sepsis or within 24 h of admission); (3) Studies with at least one of the following outcomes: all-cause mortality (in-hospital, 28-day, or 90-day), length of hospital stay (days), length of ICU stay (days), or duration of invasive mechanical ventilation (days); (4) Studies reporting sufficient data to calculate or extract an effect estimate (odds ratio [OR], hazard ratio [HR], or mean difference [MD]) with 95% confidence intervals (CIs) or raw data allowing its derivation; (5) Studies that reported adjusted effect measures controlling for key confounders (e.g., age, illness severity).

#### Exclusion criteria

Exclusion criteria were as follows: (1) Studies in pediatric populations (< 18 years); (2) Animal or *in vitro* studies; (3) Case reports or small case series (< 10 patients); (4) Studies without a comparator group (non-frail/non-sarcopenic); (5) Reviews, editorials, or conference abstracts without full data.

Where multiple publications reported overlapping cohorts, the largest or most recent dataset was selected. For the purposes of synthesis, studies of sarcopenia and frailty were grouped separately when estimating pooled effects but combined when reporting overall study characteristics.

### Information sources (databases searched)

Two reviewers performed a comprehensive search of the following electronic bibliographic databases from inception through 31 May 2025: PubMed/MEDLINE, Embase, CINAHL (Cumulative Index to Nursing and Allied Health Literature), Scopus, Web of Science Core Collection, and Cochrane Library (Cochrane Central Register of Controlled Trials). In addition, clinical trial registries (ClinicalTrials.gov; WHO International Clinical Trials Registry Platform) and gray literature (OpenGrey, conference proceedings via Embase, and institutional repositories) were reviewed. Reference lists of all included articles and relevant systematic reviews were hand-searched to identify any additional studies. All sources were last consulted on 31 May 2025.

### Search strategy

A medical librarian assisted in developing and refining the search strategies. Search terms combined controlled vocabulary (MeSH, Emtree) and keywords for “sepsis,” “septic shock,” “sarcopenia,” “frailty,” and the outcomes of interest (“mortality,” “length of stay,” “intensive care,” “mechanical ventilation”). Equivalent strategies translated to Emtree terms for Embase, CINAHL headings, and keyword combinations were applied to each database without date or language filters beyond English. No filters for study design were imposed, but conference abstracts and non-peer-reviewed materials were excluded at the screening stage. All search queries, limits, and date stamps are provided in Appendix A.

### Selection process

All records retrieved from the database searches were exported into a reference-management software (EndNote 20, Clarivate Analytics). Duplicates were removed automatically and then verified manually. Two reviewers independently screened titles and abstracts against the eligibility criteria, marking each record as “include,” “exclude,” or “unsure.” Full texts were retrieved for all studies deemed “include” or “unsure,” and these full-text articles were again reviewed independently by both reviewers. Disagreements at any stage were resolved by consensus. The study selection process was documented in a PRISMA flow diagram showing the number of records identified, screened, assessed for eligibility, and included, with reasons for exclusion at the full-text stage (24).

### Data collection process

A standardized data-extraction form was developed and piloted on a subset of five studies to ensure consistency. Two reviewers independently extracted data from each included full-text article; any discrepancies were reconciled through discussion. Extracted items included:

*Study identification*: First author, publication year, country, and study design (prospective vs. retrospective, single-center vs. multicenter).

*Population characteristics:* Sample size overall, number with sepsis/septic shock, mean or median age (± SD or IQR), sex distribution, and primary diagnosis.

*Exposure assessment:* Definition and measurement of sarcopenia (e.g., measurement modality, skeletal muscle index threshold) or frailty (e.g., frailty instrument used, cutoff values). The timing of exposure measurement relative to sepsis onset was recorded.

*Outcomes:* Mortality (in-hospital, 28-day, 90-day, or ICU mortality), length of hospital stay (days), length of ICU stay (days), and duration of mechanical ventilation (days). Where multiple timepoints were reported (e.g., 30-day vs. 90-day mortality), the earliest clinically relevant measure was prioritized for pooling; sensitivity analyses used alternative timepoints when available.

*Effect estimates:* Unadjusted and adjusted effect sizes (OR, HR, or MD) with corresponding 95% CIs. For continuous outcomes, mean (± SD) or median (IQR) values for sarcopenic/frail versus non-sarcopenic/frail groups were recorded. When only the median (IQR) was reported, the mean and standard deviation (SD) were approximated using validated methods.

*Covariates and adjustments*: Variables included in multivariable models (e.g., age, sex, Sequential Organ Failure Assessment [SOFA] score) and details of model selection.

*Risk of bias information*: Information needed to complete the risk of bias assessment tools for each study.

When data were missing or unclear, the corresponding authors were contacted via email, and two attempts were made, spaced 2 weeks apart, to request additional information or raw data. Studies that did not provide the necessary data after two attempts were excluded from the quantitative synthesis but were discussed narratively.

### Outcomes

Primary Outcomes included (1) Mortality: Dichotomous outcome (dead vs. alive) at any reported timepoint (in-hospital, 28-day, or ICU, etc.,); (2) Length of Hospital Stay: Number of days from admission to hospital discharge (mean ± SD or median [IQR]); (3) Length of ICU Stay: Number of days from ICU admission to ICU discharge (mean ± SD or median [IQR]); (4) Duration of Mechanical Ventilation: Number of days patients required invasive mechanical ventilation (mean ± SD or median [IQR]).

Secondary outcomes included: (1) Sarcopenia Definition: CT-scan muscle cross-sectional area at L3 (cm^2^/m^2^) with institution-specific sex-adjusted cut-offs, or bioelectrical impedance analysis (BIA)–derived appendicular lean mass; (2) Frailty Definition: Clinical Frailty Scale (CFS) ≥ 5, Fried Frailty Phenotype (≥ 3 criteria), or Frailty Index (≥ 0.25); (3) Covariates: Age, sex, primary comorbidities (e.g., diabetes, chronic kidney disease), severity scores (SOFA, APACHE II), nutritional status (e.g., BMI, malnutrition markers), and inflammatory markers (e.g., C-reactive protein, procalcitonin) if included in multivariable models.

Any assumptions regarding the conversion of median to mean or the transformation of effect estimates were documented in a dedicated log.

### Risk of bias assessment

Study quality was appraised using the Newcastle–Ottawa Scale (NOS) for cohort studies (25). The NOS evaluates three broad domains: (1) Selection of the exposed and non-exposed cohorts (maximum of four points), assessing representativeness of the sarcopenic/frail cohort, selection of the non-sarcopenic/non-frail comparison group, ascertainment of exposure (e.g., validated CT-based muscle measurement or established frailty assessment), and confirmation that the outcome of interest (e.g., mortality, length of stay) was not present at baseline; (2) Comparability of cohorts (maximum of two points), examining whether studies controlled for key confounders such as age and illness severity (SOFA or APACHE II); and (3) Outcome assessment (maximum of three points), which considers how outcomes were ascertained (e.g., via medical record review), adequacy of follow-up duration for each outcome (e.g., 28-day mortality), and completeness of follow-up (e.g., percentage lost to follow-up). Two reviewers independently assigned NOS stars for each study; discrepancies were resolved by consensus. Studies scoring seven to nine stars were classified as “low” risk of bias, four to six as “moderate,” and fewer than four as “high” risk. No automation tools were employed; all judgments were made by direct examination of study methods and reported results.

### Effect measures

For each outcome, the following effect measures were specified: (1) Mortality: Pooled ORs with 95% CIs comparing sarcopenic/frail versus non-sarcopenic/non-frail groups; (2) Length of Stay (Hospital or ICU) and Duration of Mechanical Ventilation: Pooled MDs with 95% CIs.

### Synthesis methods

All included studies were categorized based on the exposure type (sarcopenia vs. frailty), outcome reported, and timing of outcome measurement. For each outcome domain (mortality, length of hospital stay, ICU stay, mechanical ventilation duration), all studies providing the same effect measure at comparable timepoints were identified.

For continuous outcomes, means and SDs were used to estimate the pooled effect size. For mortality, event counts were extracted to compute ORs. Forest plots were generated for each meta-analysis, displaying individual study effect estimates with 95% CIs, study weights, and overall pooled estimates. All numerical results, including heterogeneity statistics (*I*^2^, τ^2^, Cochran’s Q), were tabulated in summary tables. Due to anticipated clinical and methodological heterogeneity in frailty/sarcopenia definitions and care settings, a random-effects model (Der Simonian–Laird) was pre-specified for all meta-analyses. This model accounts for both within-study variance and between-study variance (τ^2^). Mortality rates were pooled using the “*metan*” package in Stata 17 (StataCorp, College Station, TX), with log-transformed estimates and inverse-variance weights. Continuous outcomes (length of hospital stay, ICU stay, mechanical ventilation duration) were pooled as weighted mean differences (WMD) using the same “*metan*” function. *p*-values < 0.05 were considered statistically significant.

Heterogeneity was assessed by Cochran’s Q (*χ*^2^) test (*p* < 0.10 indicating significant heterogeneity) and quantified with *I*^2^ statistic (*I*^2^ < 25% low; 25–75% moderate; > 75% high heterogeneity) (26). Tau-squared (τ^2^) was reported as an absolute measure of between-study variance. For outcomes with ≥ 10 studies (mortality and length of hospital stay), we assessed small-study effects and publication bias using funnel plot visual inspection and Egger’s regression test (*p* < 0.10), indicating asymmetry.

## Results

### Search results

**Figure 1:**
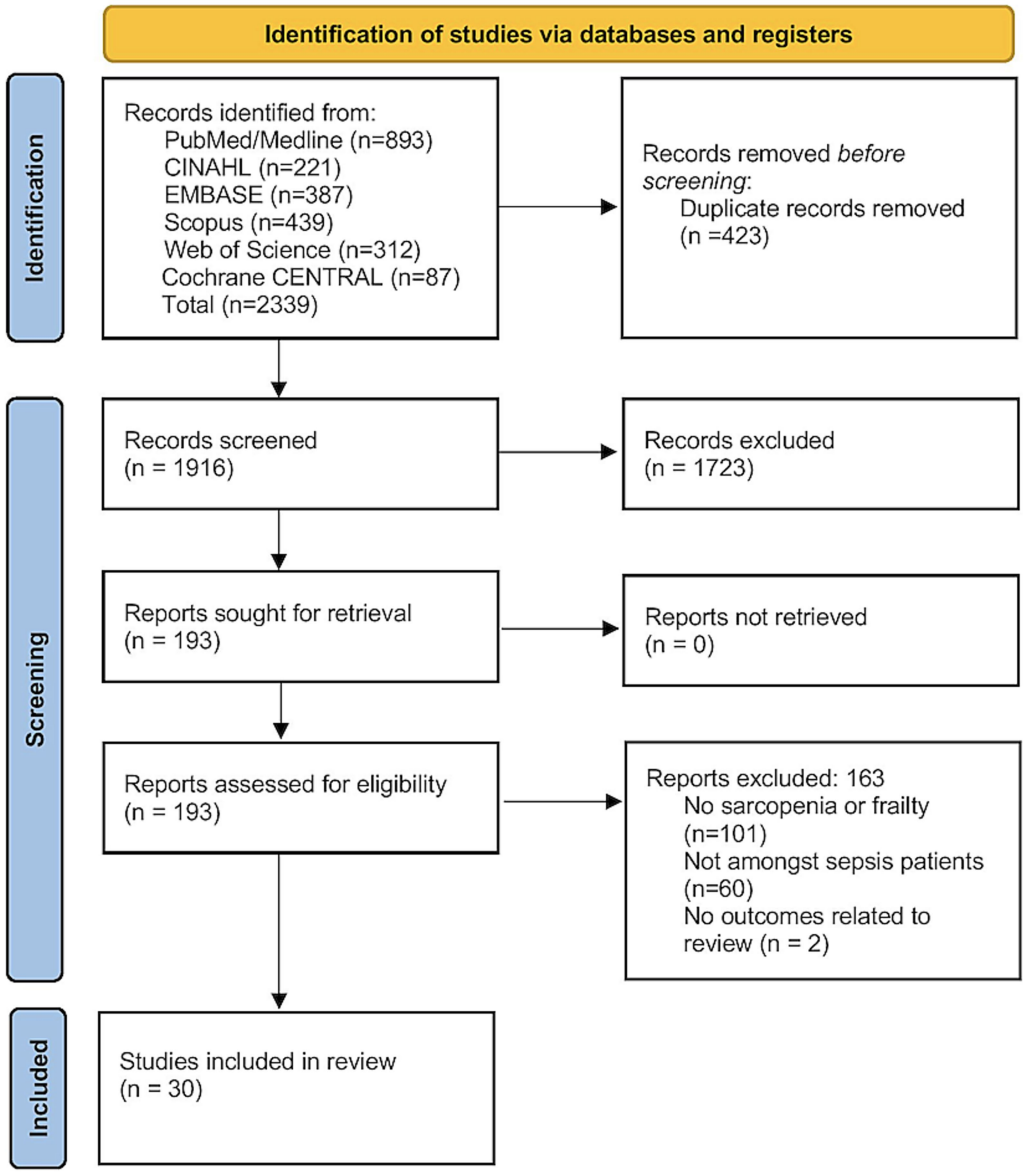
PRISMA flowchart.

Figure 1 summarizes the study selection process. A total of 2,339 records were identified through database searches, of which 423 duplicates were removed before screening. After title and abstract review of 1,916 unique records, 1,723 were excluded. Full texts of 193 articles were assessed for eligibility, leading to the exclusion of 163 reports. Of them, 101 did not assess sarcopenia or frailty, 60 were not conducted in sepsis populations, and 2 lacked relevant outcomes. Ultimately, 30 studies met all criteria and were included (18, 27–54).

### Characteristics of the included studies

Table 1 summarizes the characteristics of the 30 studies assessing sarcopenia or frailty in patients with sepsis or septic shock and their association with various mortality endpoints. These investigations were conducted across nine countries: China (*n* = 8), Korea (*n* = 8), USA (*n* = 4), Japan (*n* = 4), Netherlands (*n* = 2), and one study each from France, Italy, Australia, and India. Twenty-one studies used a retrospective cohort or observational design, while nine were prospective cohort studies. Sample sizes varied widely, from as few as 47 participants to as many as 21,338, and mean patient ages spanned from 49.7 ± 12.6 years to 89.5 ± 9 years.

**Table 1:** Characteristics of the included studies (*N* = 30).

S. No	Study identifier	Country	Study design	Participant details	Sample size	Sarcopenia or frailty criteria	Age in terms of Mean (SD)	Outcome assessed	Risk of bias
1	Wang et al. (2024) (27)	China	Retrospective Cohort Study	Patients with sepsis-3 at ICU	115	Based on Psoas Muscle	75.6 ± 4	28-day mortality	Low
2	Kim et al. (2025) (28)	Korea	Retrospective Study	Patients with Sepsis, AKI and underwent CRRT	618	Based on SMI cut-off from CT scan	68 ± 5.4	28-day mortality	Low
3	Kim, 2023 (59)	Korea	Retrospective Observational Study	Patients with sepsis at ED	374	PMI < 423 mm^2^/m^2^ for males and <269mm^2^/m^2^ for females	65 ± 8	In Hospital Mortality	High
4	Oh et al. (2022) (29)	Korea	Retrospective Cohort Study	Patients with septic shock	905	SMI has <45.4 cm^2^/m^2^ in men and 34.4 cm^2^/m^2^ in women	68.3 ± 14	1-year mortality	High
5	Darden et al. (2023) (31)	USA	Retrospective Cohort Study	Patients are critically ill in ICU	150	SMI has ≤ 41.6 cm^2^/m^2^ for males and ≤ 32.0 cm^2^/m^2^ for females	75 ± 8	In Hospital Mortality	Moderate
6	Herault et al. (2023) (30)	France	Retrospective Cohort Study	Patients with sepsis or septic shock in ICU	114	CT performed based on L3 and T12	58 ± 6	In Hospital Mortality	Moderate
7	Cox et al. (2021) (32)	USA	Prospective Cohort Study	Patients with sepsis-3 at ICU	47	SMI (CT) has <52.4cm^2^/m^2^ for male and <38.5cm^2^/m^2^ for female	53.1 ± 12	30-day mortality	Low
8	Ji et al. (2018) (33)	China	Retrospective Cohort Study	Patients with sepsis-2 at ICU	126	SMI (CT) has <40.8cm^2^/m^2^ for male and < 34.9 cm^2^/m^2^ for female	67.8 ± 8	30-day mortality	Low
9	Kim et al. (2019) (34)	Korea	Retrospective Cohort Study	Patients with sepsis-3 at ICU	516	SMI (CT) has <55cm^2^/m^2^ for male and <39cm^2^/m^2^ for female	68.6 ± 14	1-year mortality	High
10	Ketenci et al. (2018) (35)	Japan	Retrospective Cohort Study	Patients with sepsis-3 at ICU	191	SMI (CT) has <80% of predicted value	71.8 ± 9	In Hospital Mortality	High
11	Leeet al. (2018) (36)	Korea	Retrospective Cohort Study	Patients with sepsis-3 at ED	274	SMI (CT) has <545mm^2^/m^2^ for male and < 385 mm^2^/m^2^ for female	70.9 ± 12.5	28-day mortality	High
12	Lucidi et al. (2018) (37)	Italy	Retrospective Cohort Study	Patients with sepsis-1 at ICU	74	MAMC has <95% of predicted value	49.7 ± 12.6	In Hospital Mortality	Moderate
13	Okada et al. (2021) (38)	Japan	Retrospective Cohort Study	Patients with sepsis-3 at ICU	171	SMI (CT) has T3:T1 of psoas index	NA	1-year mortality	Moderate
14	Seo et al. (2019) (39)	Korea	Retrospective Cohort Study	Patients with sepsis-3 at the ED	175	SMI (CT) has <53cm^2^/m^2^ for a BMI of 25 kg/m^2^ or more for male and < 41 cm^2^/m^2^ regardless of the BMI for female	65 ± 12	28-day mortality	Moderate
15	Shibahashi et al. (2017) (40)	Japan	Retrospective Cohort Study	Patients with sepsis-3 in the ICU	150	SMA (CT) has <45.2cm^2^ for male and <39.0cm^2^ for female	75 ± 8	In Hospital Mortality	High
16	Baggerman et al. (2020) (41)	Netherlands	Retrospective Cohort Study	Patients with sepsis in the ICU	48	Based on SMI CT scan	69.5 ± 12	In Hospital Mortality	Low
17	Cho et al. (2019) (42)	Korea	Retrospective Cohort Study	Patients are critically ill in the ICU	94	Based on TPA from CT scan	60.2 ± 8.9	1-year mortality	Low
18	Ebbeling et al. (2013) (43)	USA	Prospective Cohort Study	Patients under trauma care in the ICU	180	Based on TPA from CT scan	79 ± 15	In Hospital Mortality	Low
19	Joyce et al. (2020) (45)	Australia	Retrospective Observational Study	Patients are critically ill in the ICU	279	Based on SMA from CT	67.8 ± 14.8	30-day mortality	Moderate
20	Kou et al. (2019) (46)	China	Retrospective Cohort Study	Patients are critically ill in the ICU	96	Based on TPA from CT scan	67.5 ± 17	In Hospital Mortality	Moderate
21	de Hoogt et al. (2017) (44)	Netherlands	Retrospective Cohort Study	Patients are critically ill in the ICU	139	Based on SMI CT scan	NA	In Hospital Mortality	Low
22	Li et al. (2023) (53)	China	Prospective Cohort Study	Older patients with intra-abdominal sepsis	464	Based on SMI CT scan	75 ± 7	In Hospital Mortality	Low
23	Li et al. (2023) (54)	China	Prospective Cohort Study	Older patients with sepsis in the emergency	443	Based on SMI CT scan	78 ± 6	90-day mortality	Moderate
24	Dong et al. (2023) (18)	China	Prospective Cohort Study	Older patients with sepsis in ICU	54	According to clinical fraility scale	89.5 ± 9	In Hospital Mortality	Low
25	Ding et al. (2024) (47)	China	Prospective Cohort Study	Patients with septic shock in ICU	3,606	Based on FL-Lab Index	70.2 ± 16	1-year mortality	Low
26	Lee et al. (2022) (48)	Korea	Prospective Cohort Study	Patients diagnosed with sepsis	936	Clinical frailty score between 1 to 4	70 ± 14.6	In Hospital Mortality	Moderate
27	Li et al. (2024) (49)	China	Retrospective Cohort Study	Patients diagnosed with sepsis in the ICU	21,338	Based on modified frailty index ≥3	73.16 ± 18	In Hospital Mortality	Moderate
28	Mahalingam et al. (2019) (50)	USA	Prospective Cohort Study	Patients diagnosed with sepsis as hospitalized for a serious infection with ≥2 system inflammatory response syndrome criteria.	6,988	Frailty as the presence of at least 2 frailty indicators (weakness, exhaustion, and low physical activity)	66.2 ± 10	30-day mortality	Low
29	Matsuda et al. (2020) (51)	Japan	Retrospective Cohort Study	Patients with sepsis on Mechanical Ventilation who underwent protocol-based weaning	99	Clinical Frailty Scale score 4 or more	78 ± 16	In Hospital Mortality	Low
30	Murlidharan et al. (2022) (52)	India	Prospective Cohort Study	Patients diagnosed with sepsis	50	Based on Frailty Index	71.3 ± 6.5	90-day mortality	Moderate

ED, emergency department; ICU, intensive care unit; SMI, skeletal muscle index; SMA, skeletal muscle area; MAMC, midarm muscle circumference; CT, computed tomography; BMI, body mass index; TPA, total psoas muscle area.

Definitions of sarcopenia or frailty differed markedly between studies. The majority (20/30) relied on CT-derived muscle metrics, such as skeletal muscle index (SMI), psoas muscle index (PMI), skeletal muscle area (SMA), or total psoas area (TPA), using gender- and, in some cases, BMI-specific cut-offs. Others employed anthropometric measures (e.g., mid-arm muscle circumference), clinical frailty scales, modified frailty indices (≥3), or composite laboratory-based indices. Mortality outcomes were also heterogeneous: 28-day mortality was reported in four studies, 30-day mortality in five, in-hospital mortality in 14, one-year mortality in five, and 90-day mortality in two. Risk-of-bias assessments classified 13 studies as low risk, 11 as moderate risk, and 6 as high risk.

### Impact of sarcopenia or frailty on mortality

**Figure 2:**
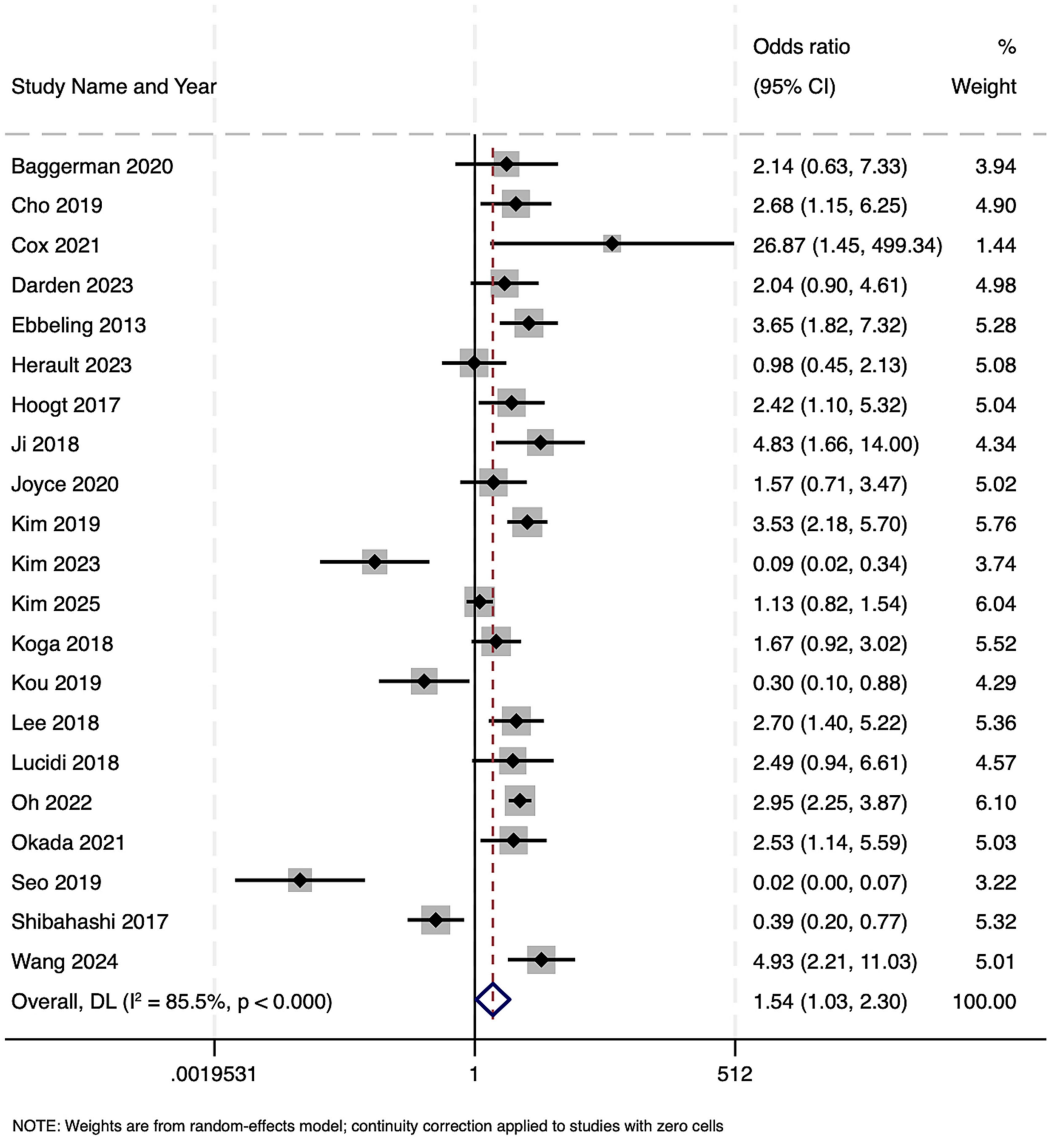
Forest plot showing the impact of sarcopenia on mortality among patients with sepsis.

Across 21 studies encompassing 4,836 patients with sepsis or septic shock, the presence of sarcopenia was associated with significantly increased odds of in-hospital mortality (OR 1.54, 95% CI 1.03–2.30; *z* = 2.12, *p* = 0.034) (Figure 2). However, there was marked between-study heterogeneity (Cochran’s Q = 137.89, df = 20, *p* < 0.001; *I*^2^ = 85.5%), indicating substantial variability in effect estimates across cohorts. The estimated between-study variance (τ^2^) was 0.6640. Funnel plot was symmetrical (Supplementary Figure 1) and Egger’s test was non-significant (*p* = 0.28), indicating no publication bias. Due to variation in the timepoint of mortality assessment, subgroup analysis was done. Significant increases were seen for 1-year mortality (OR = 3.05, 2.43–3.83; *I*^2^ = 0%), in-hospital mortality (OR = 1.70, 1.15–2.52; *I*^2^ = 8.3%), and 30-day mortality (OR = 3.59, 1.07–12.11; *I*^2^ = 62.3%). No clear association was observed for pooled 28-day mortality (OR = 2.33, 0.94–5.76; *I*^2^ = 86.3%). Between-subgroup heterogeneity was significant (*p* < 0.001).

**Figure 3:**
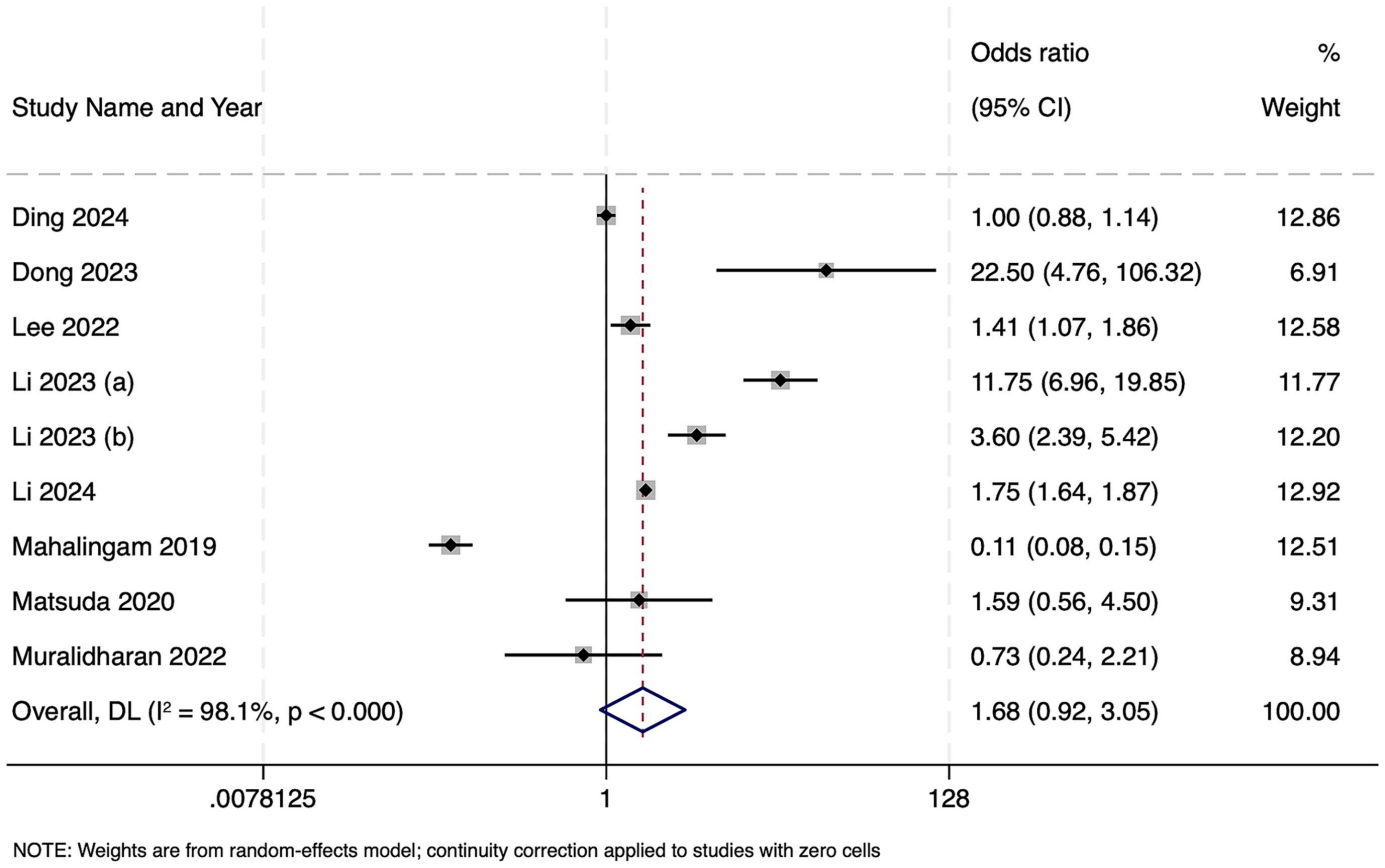
Forest plot showing the impact of frailty on mortality among patients with sepsis.

Frailty was reported in nine studies with 33,978 participants. The results showed that frailty was associated with a non-significant trend toward higher mortality in sepsis (OR 1.68, 95% CI 0.92–3.05; *z* = 1.69, *p* = 0.091) (Figure 3). There was very high between-study heterogeneity (Cochran’s Q = 419.59, df = 8, *p* < 0.001; *I*^2^ = 98.1%), and the estimated between-study variance (τ^2^) was 0.7202. Publication bias assessment was not possible due to limited number of studies and hence, the results should be interpreted with caution.

### Impact of sarcopenia or frailty on length of hospital stay

**Figure 4:**
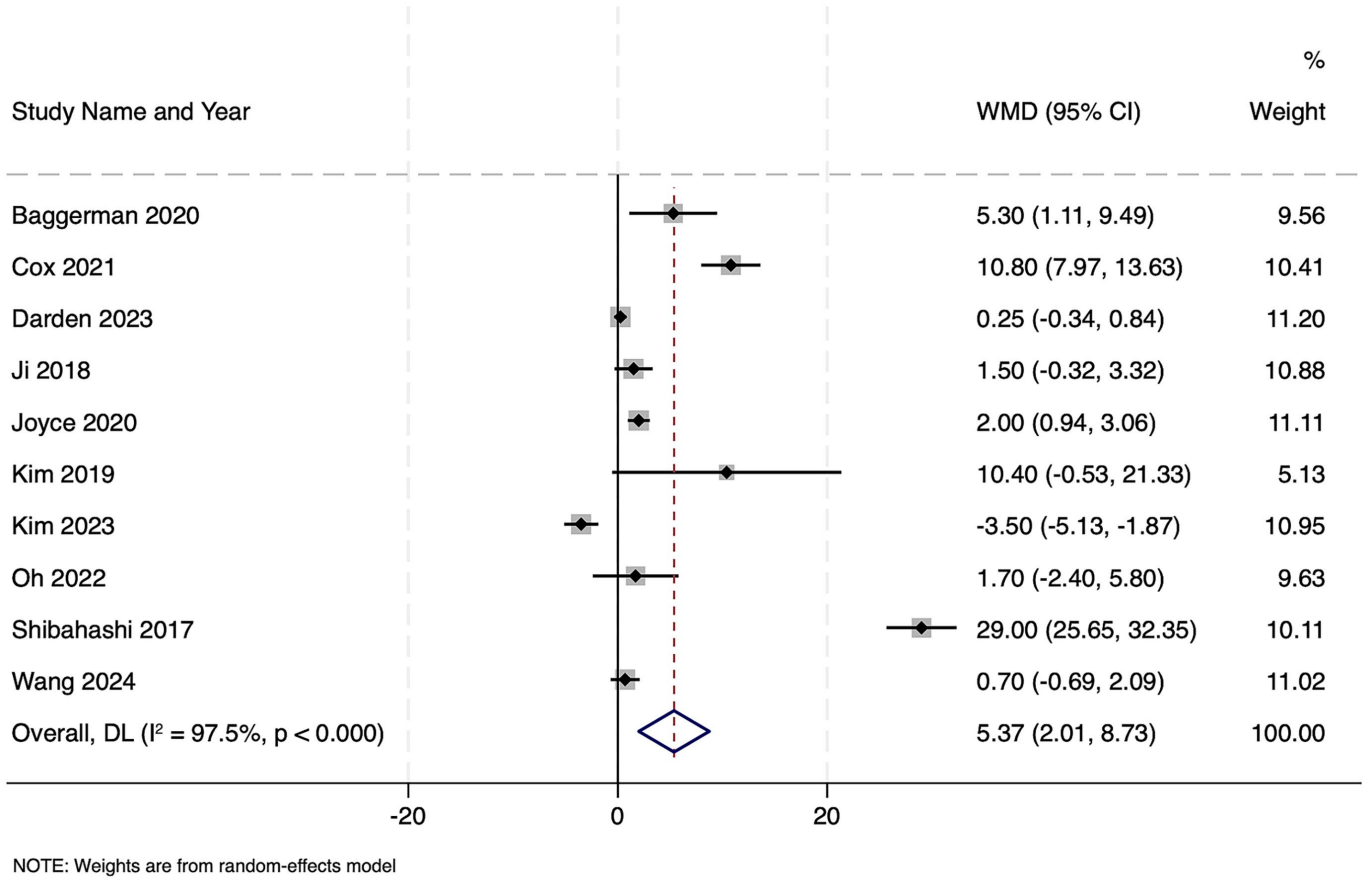
Forest plot showing the impact of sarcopenia on length of hospital stay among patients with sepsis.

Ten studies reported on 2,710 patients with sepsis who were classified as sarcopenic. Compared to non-sarcopenic patients, the presence of sarcopenia was associated with a significantly longer hospital stay, with the pooled WMD of 5.37 days (95% CI 2.01–8.73; *z* = 3.13, *p* = 0.002), indicating that sarcopenic patients remained hospitalized over 5 days longer on average than their non-sarcopenic counterparts (Figure 4). Heterogeneity was extremely high (Cochran’s Q = 360.52, df = 9, *p* < 0.001; *I*^2^ = 97.5%), with τ^2^ = 26.16. Funnel plot was symmetrical (Supplementary Figure 2) and Egger’s test was non-significant (*p* = 0.13), indicating no publication bias.

**Figure 5:**
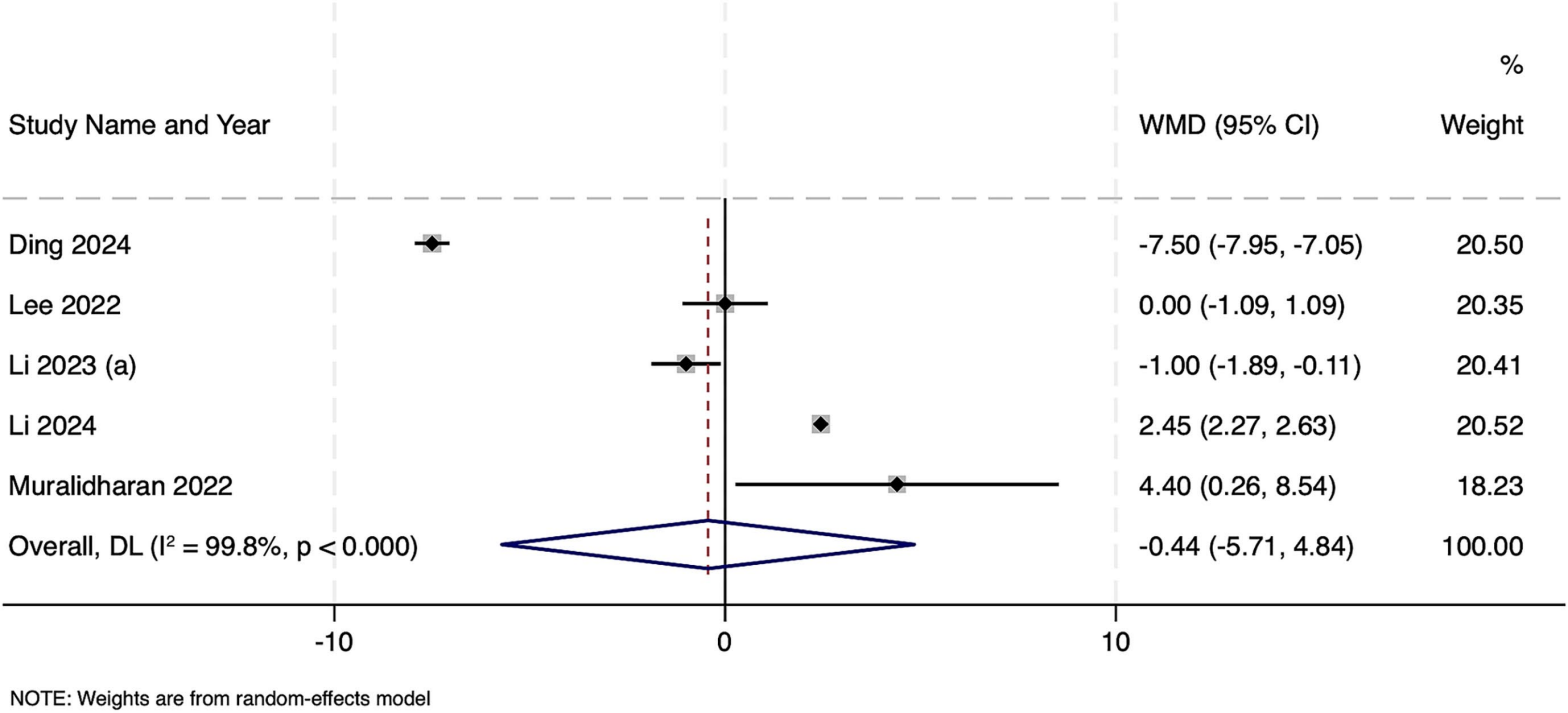
Forest plot showing the impact of frailty on length of hospital stay among patients with sepsis.

Five studies with 26,394 septic patients reported outcomes, categorized by frailty status. Frail patients did not have a statistically significant difference in hospital length of stay compared to non-frail patients (WMD − 0.44 days, 95% CI − 5.71 to 4.84; *z* = −0.16, *p* = 0.87) (Figure 5). Extreme heterogeneity was observed (Cochran’s Q = 1678.36, df = 4, *p* < 0.001; *I*^2^ = 99.8%; τ^2^ = 35.28). Publication bias assessment was not possible due to limited number of studies and hence, the results should be interpreted with caution.

### Impact of sarcopenia or frailty on length of ICU stay

**Figure 6:**
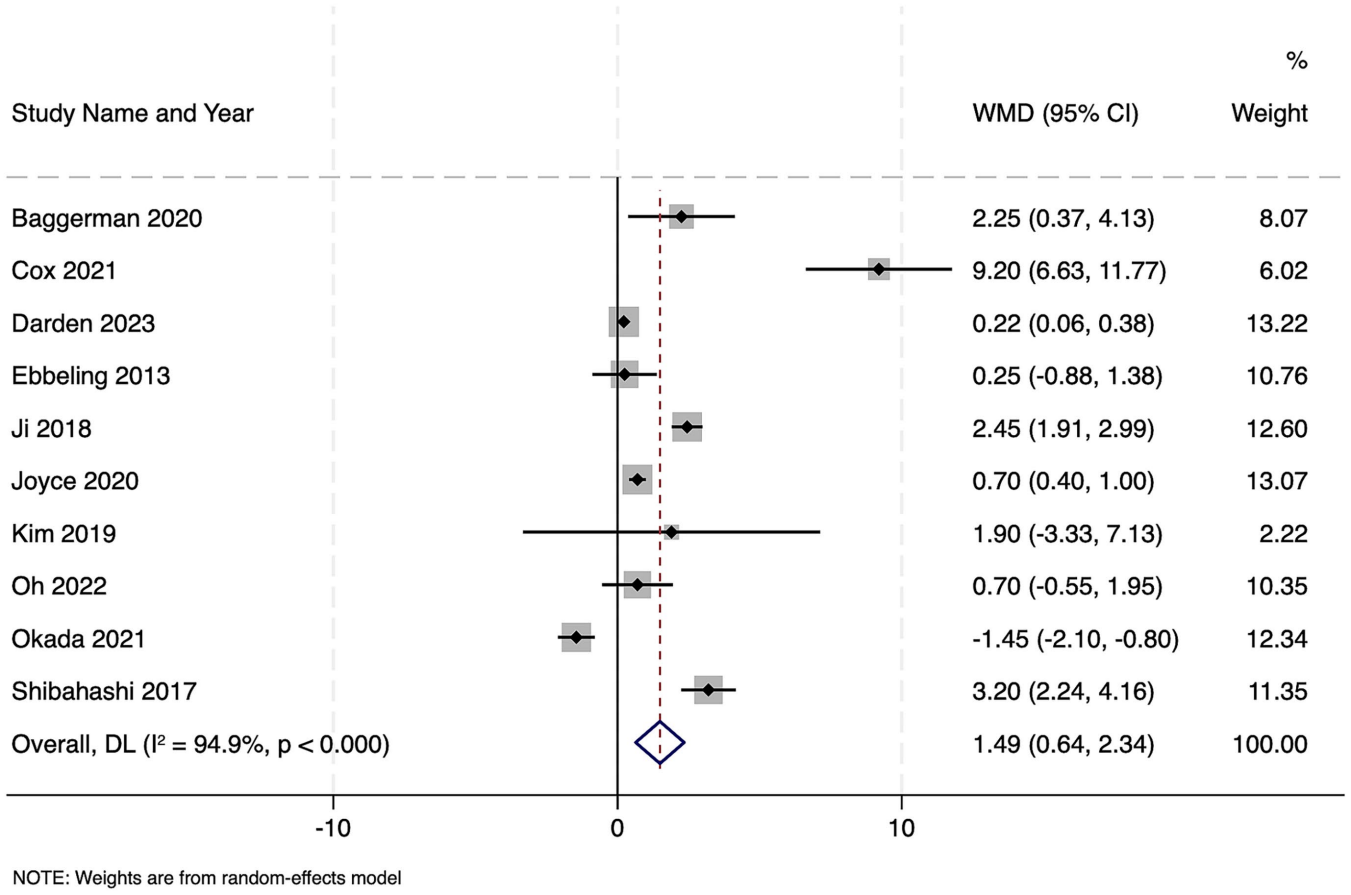
Forest plot showing the impact of sarcopenia on length of ICU stay among patients with sepsis.

Across 10 studies comprising 2,572 septic patients, sarcopenic individuals experienced significantly longer ICU stays (WMD 1.49 days; 95% CI 0.64–2.34; *z* = 3.42, *p* = 0.001) (Figure 6). However, heterogeneity was extreme (Cochran’s Q = 175.08, df = 9, *p* < 0.001; *I*^2^ = 94.9%; τ^2^ = 1.4264). Funnel plot was symmetrical (Supplementary Figure 3) and Egger’s test was non-significant (*p* = 0.16), indicating no publication bias.

**Figure 7:**
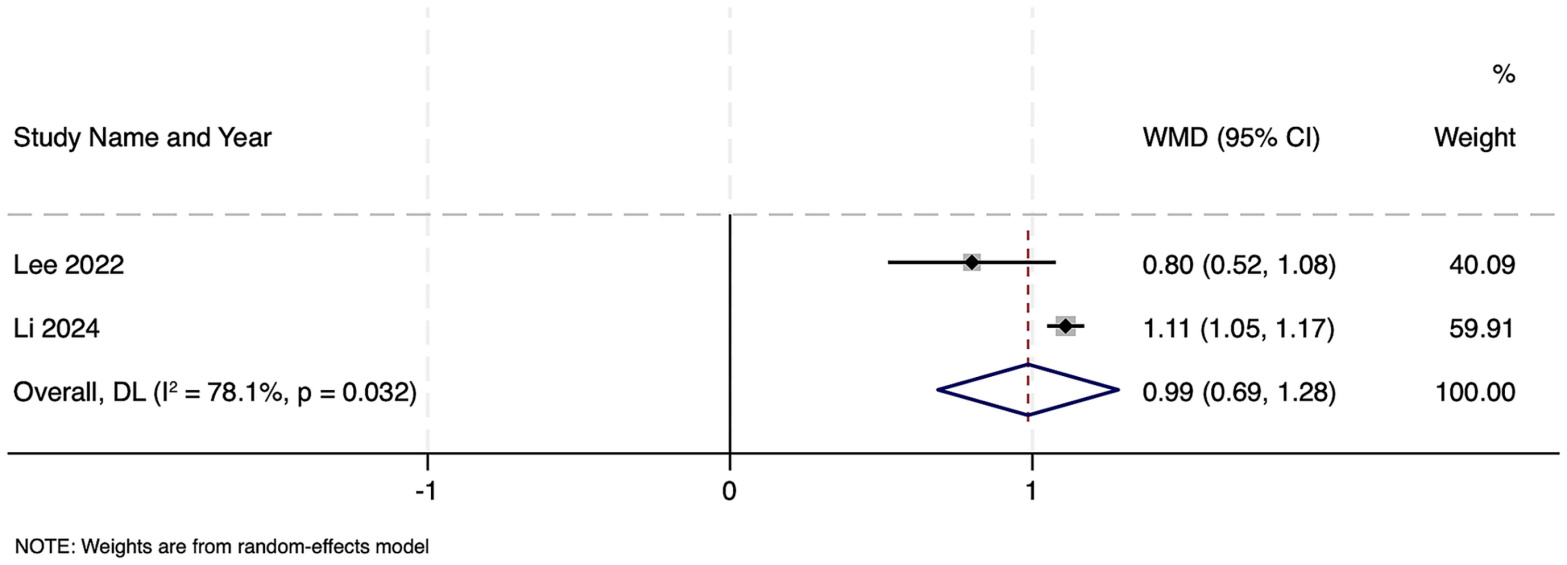
Forest plot showing the impact of frailty on length of ICU stay among patients with sepsis.

Among two large cohorts of 22,274 septic patients, frailty was associated with significantly longer ICU stay, with a pooled WMD of 0.99 days (95% CI: 0.69–1.28; *z* = 6.49, *p* < 0.001). These results indicate that frailty conferred nearly one additional day in the ICU (Figure 7). Although heterogeneity was substantial (Cochran’s Q = 4.57, df = 1, *p* = 0.032; *I*^2^ = 78.1%; τ^2^ = 0.0375). Publication bias assessment was not possible due to limited number of studies and hence, the results should be interpreted with caution.

### Impact of sarcopenia on duration of mechanical ventilation

**Figure 8:**
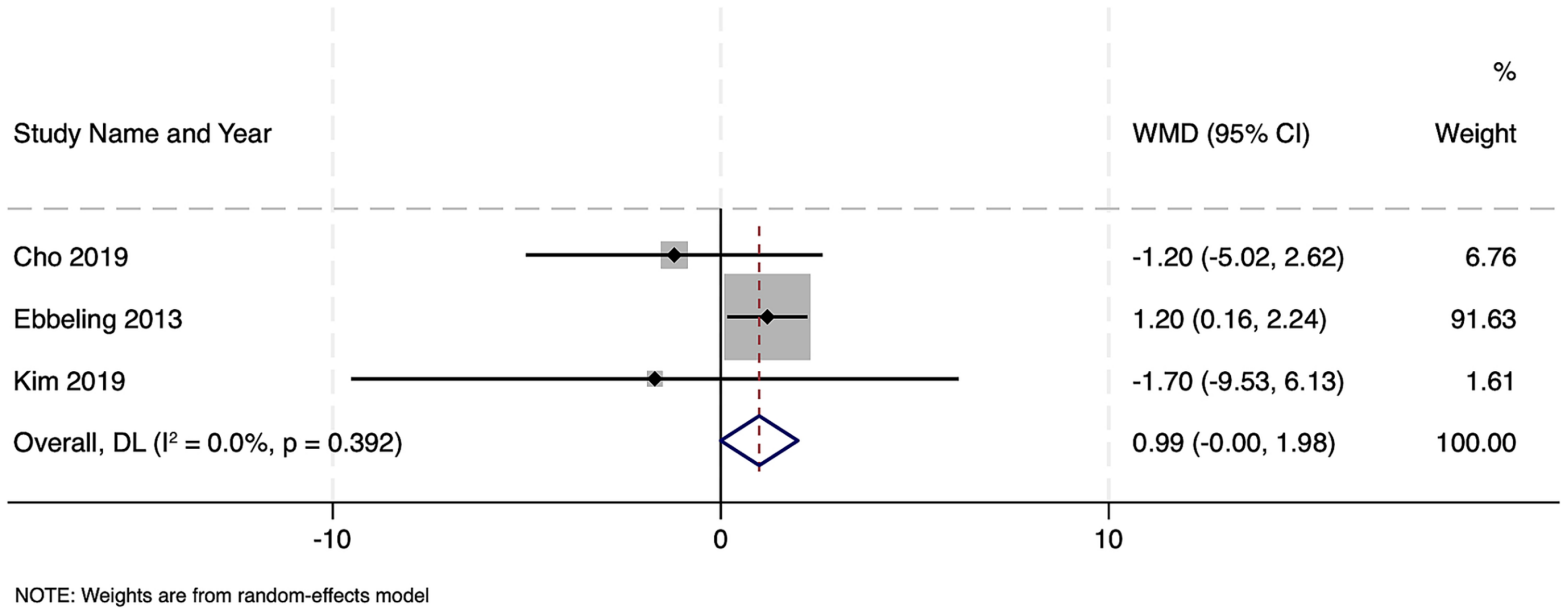
Forest plot showing the impact of sarcopenia on duration of mechanical ventilation among patients with sepsis.

Among three studies totaling 790 patients, sarcopenic individuals required an average of 0.99 more days of mechanical ventilation than non-sarcopenic patients, a difference that approached but did not reach statistical significance (WMD 0.99 days, 95% CI –0.003 to 1.99; *z* = 1.955, *p* = 0.051) (Figure 8). There was no evidence of between-study heterogeneity (Cochran’s Q = 1.87, df = 2, *p* = 0.392; *I*^2^ = 0.0%; τ^2^ = 0.000). Publication bias assessment was not possible due to the limited number of studies and hence, the results should be interpreted with caution. No studies reported the impact of frailty on the duration of mechanical ventilation.

## Discussion

This comprehensive meta-analysis of 30 observational studies showed that sarcopenia is consistently associated with worse outcomes across multiple clinically important domains, such as higher in-hospital mortality, significantly longer hospital and ICU stays, as well as a trend toward prolonged mechanical ventilation. Frailty, by contrast, demonstrated a nonsignificant trend toward increased mortality and length of hospital stay but was associated with a nearly one-day longer ICU admission in the two large cohorts available for that outcome. Taken together, these findings underscore sarcopenia as a stronger and more consistent prognostic factor than frailty in sepsis. In contrast, frailty appears to exert a more modest and less consistent effect on overall survival and hospital length of stay. For ICU length of stay, both sarcopenia and frailty were associated with significantly prolonged admission, indicating that physical vulnerability broadly predicts greater resource utilization in critical care.

Our results align closely with those of prior single-center and registry-based studies, suggesting that low muscle mass increases vulnerability to critical illness (55). Early observational reports showed that CT-derived muscle depletion predicted mortality among septic patients, which is consistent with the pooled OR (1.54) in this study, substantiated across heterogeneous settings and measurement methods. Prior narrative reviews have highlighted sarcopenia’s association with prolonged ventilator dependence (42, 43). This meta-analysis further confirms a trend toward nearly one additional ventilation day in sarcopenic cohorts, though the small number of studies limited statistical significance. The magnitude of the effect of sarcopenia on the length of hospital stay in this analysis (WMD + 5.37 days) exceeds estimates from some earlier cohort reports that suggested incremental increases of 2–3 days (40, 45). Such discrepancy may be due to the inclusion of larger, multi-center datasets that better capture real-world variation in this study.

In contrast, the literature on frailty in sepsis has been more fragmented, with individual studies reporting mixed results. Some single-center cohorts found that frailty doubled the mortality risk (48, 49), while others observed minimal or no association (47, 52). This inconsistency was further confirmed by the pooled estimate (OR 1.68, *p* = 0.091) of this study. Although point estimates favored higher mortality, wide confidence intervals and extreme heterogeneity (*I*^2^ = 98.1%) rendered the association nonsignificant. This variability may reflect differences in frailty instruments (e.g., Clinical Frailty Scale versus Frailty Index), thresholds for “frail” classification, and underlying comorbidity burdens. Similarly, studies focusing on hospital length of stay in frail septic patients have reported highly heterogeneous durations of hospitalization (47, 52), further confirmed by the results of this study. However, the consistent finding that frailty lengthens ICU stay by nearly 1 day supports prior single-center work suggesting frailty impairs weaning and prolongs organ support (48, 49).

Overall, these findings suggest that sarcopenia exerts a more robust and reproducible effect across multiple sepsis outcomes than frailty. It is plausible that direct quantification of muscle mass is both objective and more closely linked to metabolic reserve, whereas frailty encompasses broader domains (comorbidity, cognition, social factors) that may variably influence care decisions, care limitations, and surrogate endpoints.

The pathophysiology underlying the deleterious impact of sarcopenia on sepsis outcomes is multifactorial. Muscle tissue serves both as a metabolic reservoir and a source of amino acids that support gluconeogenesis and immunomodulatory protein synthesis during catabolic stress (56). In sarcopenic patients, therefore, diminished lean mass leads to more profound metabolic instabilities, poorer immune responses, and impaired anabolic recovery after septic insult (43, 45). Moreover, respiratory muscle weakness—common in sarcopenia—promotes delayed ventilator weaning (57), which likely accounts for the nearly one-day trend toward prolonged mechanical ventilation noted in this study.

Extended hospital and ICU stays among sarcopenic patients may also reflect slower rehabilitation and a higher incidence of nosocomial complications, such as nosocomial infections or delirium, which amplify the length of admission. Prolonged immobilization further exacerbates catabolism, creating a vicious cycle (58). In comparison, frailty, as a broader geriatric syndrome, encompasses not only diminished muscle mass but also cognitive impairment, multimorbidity, and reduced physiologic reserve across multiple organ systems. These factors collectively predict worse outcomes, yet may also lead to earlier treatment limitation or palliative approaches, attenuating the apparent effect on hospital stay and mortality in pooled observational data.

Similarly, the impact of frailty on ICU length of stay likely arises from mechanisms such as reduced physiologic reserve and slower recovery, particularly in terms of weaning from ventilator and vasoactive support, as well as nutritional recovery. However, it is possible that heterogeneous definitions of frailty, variable censoring for early mortality, and diverse end-of-life decision-making practices likely account for the lack of a consistent association with overall mortality or hospital stay.

The major strength of this meta-analysis is that it encompasses 30 studies with over 38,000 patients from diverse geographic regions, intensive care unit (ICU) settings, and measurement modalities. By rigorously applying the PRISMA framework and utilizing random-effects models, the study provided a comprehensive and updated synthesis. The use of objective imaging or functional assessments for sarcopenia (e.g., CT-derived skeletal muscle index, handgrip strength) and validated frailty tools (e.g., Clinical Frailty Scale) across primary studies further lends credibility to the findings.

Nevertheless, the study has some limitations. First, all included studies were observational, subject to unmeasured confounding. For instance, sicker patients are more likely to undergo imaging or frailty assessment, potentially introducing selection bias. Second, heterogeneity was extreme for many outcomes, particularly frailty-related mortality (*I*^2^ = 98.1%) and sarcopenia-related length of stay (*I*^2^ = 97.5%). This may have limited confidence in the pooled estimates. Third, some outcomes had few contributing studies (e.g., mechanical ventilation duration), which restricted statistical power and precluded a robust assessment of publication bias.

The consistent finding that sarcopenia portends worse outcomes in sepsis has important practical implications for bedside management. First, early identification of low muscle mass should be incorporated into routine risk stratification upon admission to the ICU. Many centers now perform abdominal or thoracic imaging as part of the sepsis workup. The opportunistic measurement of skeletal muscle cross-sectional area on CT scans can rapidly identify patients with sarcopenia. Likewise, simple bedside assessments, such as handgrip dynamometry or ultrasound-based muscle thickness measurements, can be integrated into initial evaluations in settings without ready access to CT analysis. Recognizing sarcopenia at presentation may allow clinicians to flag high-risk patients who may benefit from more aggressive monitoring, more judicious fluid resuscitation to avoid iatrogenic overload, and earlier nutritional and physical rehabilitation interventions.

The use of computed tomography (CT) scans to assess skeletal muscle mass offers highly objective, reproducible, and validated metrics of sarcopenia, including the skeletal muscle index (SMI), psoas muscle index (PMI), and total psoas area (TPA). In the setting of sepsis, CT scans are frequently obtained for diagnostic purposes, affording an opportunity to opportunistically evaluate muscle mass without additional radiation or cost. However, the routine use of CT-based screening for sarcopenia or frailty prior to or early during sepsis admission may be limited by several practical factors. First, not all septic patients undergo abdominal imaging at presentation; in some settings, imaging may be reserved for specific clinical indications such as suspected source localization. Second, CT analysis requires trained personnel, dedicated software, and institutional protocols, which may not be widely available, especially in resource-limited environments. Third, patient factors such as hemodynamic instability can preclude CT imaging.

Alternative bedside assessment modalities, such as handgrip dynamometry, ultrasound-based muscle thickness measurement, or validated frailty screening tools (e.g., Clinical Frailty Scale), may offer more feasible options for early identification in clinical workflows. Nevertheless, whenever CT imaging is performed for sepsis diagnostic workup, integration of muscle mass assessment should be strongly considered, as it does not increase risk or cost and can rapidly stratify patients at high risk for adverse outcomes. Future studies should explore streamlined CT analysis protocols, automated image quantification, and the comparative effectiveness of imaging versus bedside screening approaches in different sepsis populations.

Although frailty demonstrated a less consistent relationship with mortality and hospital length of stay, its clear association with longer ICU admission highlights the need to assess frailty as part of shared-decision discussions. In practice, frailty screening should prompt early conversations about goals of care, anticipated prognosis, and potential limitations of aggressive interventions. Conversely, identifying frailty may also serve as a trigger for pre-emptive measures to prevent ICU-related deconditioning, including early mobilization and delirium prevention protocols, which can help attenuate the functional decline that often leads to protracted ICU stays.

Finally, the demonstration that sarcopenia, but not frailty, consistently prolongs hospital stay underscores the importance of muscle-preserving strategies in sepsis bundles. While fluid resuscitation, antibiotic timing, and hemodynamic support remain cornerstones of sepsis care, the findings of this study suggest that integrating nutritional support protocols, such as early enteral feeding enriched with protein and branched-chain amino acids, should be prioritized for patients with documented muscle depletion.

Further prospective interventional trials should evaluate whether targeted muscle-preserving and rehabilitation strategies can mitigate the excess risk associated with sarcopenia. Randomized studies comparing standard sepsis management with standard management plus early mobilization, high-protein nutritional interventions, or novel anabolic pharmacotherapies (e.g., selective androgen receptor modulators) in sarcopenic patients will help determine causality rather than mere association. Similarly, research priorities should include optimizing the timing, intensity, and modality of physiotherapy in sepsis. For example, trials comparing passive versus active mobilization or modalities such as neuromuscular electrical stimulation could clarify best practices.

Ultimately, the interplay between sarcopenia and frailty warrants further elucidation. Although both conditions frequently coexist, few studies have simultaneously measured muscle mass and frailty indices in the same cohort. Future research should evaluate whether the combination of sarcopenia and frailty exerts additive or synergistic risk, and whether interventions that target muscle mass alone may suffice to improve outcomes, or if broader geriatric-oriented interventions are needed for frail patients. Extensive multicentre observational studies that collect granular data on both domains could help disentangle these relationships and refine personalized care pathways.

## Conclusion

In patients with sepsis and septic shock, sarcopenia found to be significant predictor of worse in-hospital mortality, prolonged hospital and ICU lengths of stay, and a trend toward prolonged mechanical ventilation. While frailty demonstrates a more modest and less consistent association with mortality and hospital stay, it still confers longer ICU admissions. The findings underscore the importance of early assessment of muscle mass and the implementation of muscle-preserving strategies, nutritional optimization, early mobilization, and multidisciplinary rehabilitation in modern sepsis care bundles.

## Data Availability

Publicly available datasets were analyzed in this study. This data can be found here: PubMed, EMBASE, Scopus, CINAHL, Web of Science, and CENTRAL databases were searched from inception to May 31, 2025.

## Supplementary material

SUPPLEMENTARY FIGURE 1 Funnel plot for impact of sarcopenia on mortality.

SUPPLEMENTARY FIGURE 2 Funnel plot for impact of sarcopenia on length of hospital stay.

SUPPLEMENTARY FIGURE 3 Funnel plot for impact of sarcopenia on length of ICU stay.
