# Supplementary figures and images for: Impact of sarcopenia and frailty on outcomes of patients with sepsis or septic shock: a systematic review and meta-analysis
Source: Front Nutr. 2025 Oct 13;12:1679632. doi: 10.3389/fnut.2025.1679632 (PMC12554568; doi:10.3389/fnut.2025.1679632)

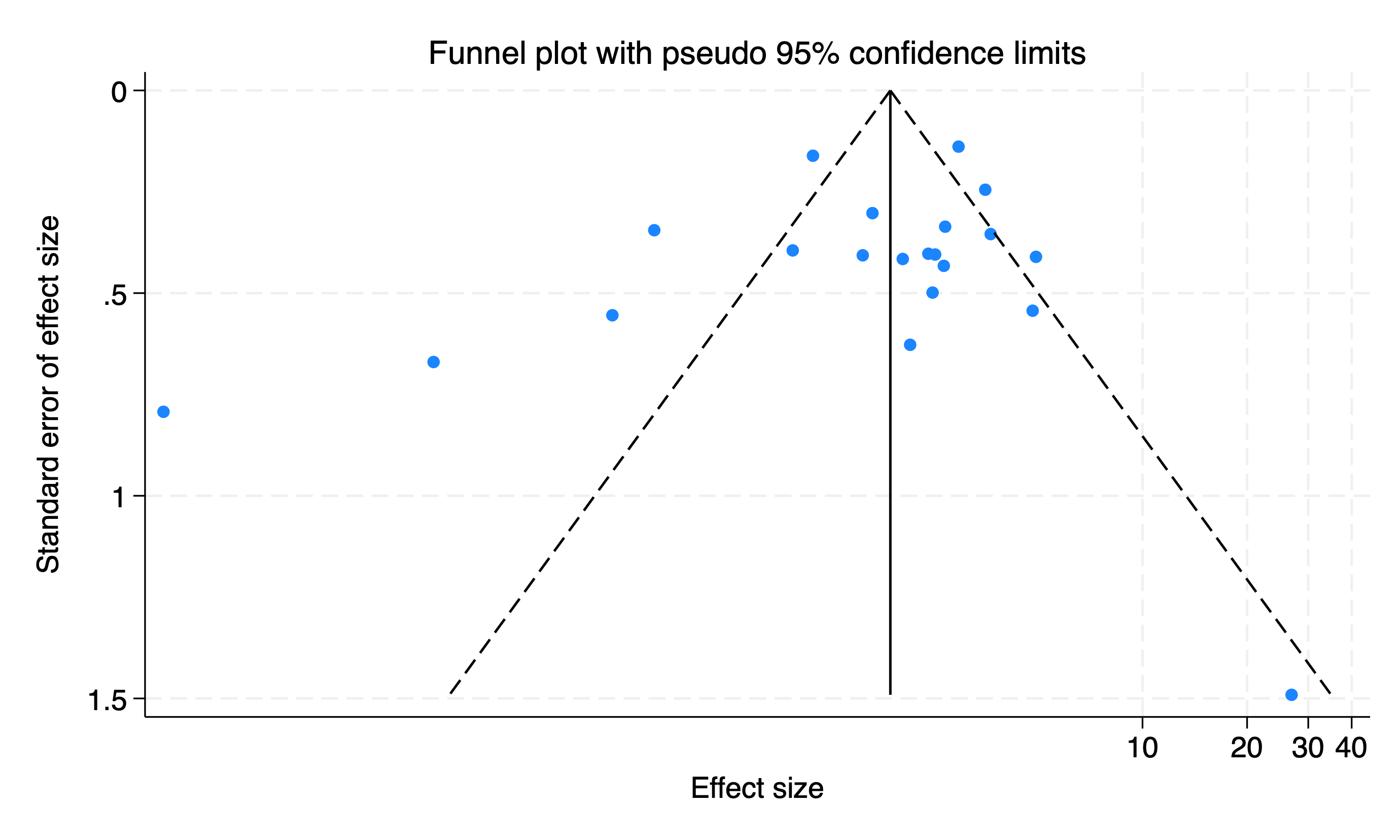

Supplement: SUPPLEMENTARY FIGURE 1 — Funnel plot for impact of sarcopenia on mortality. [file Image_1.TIF]

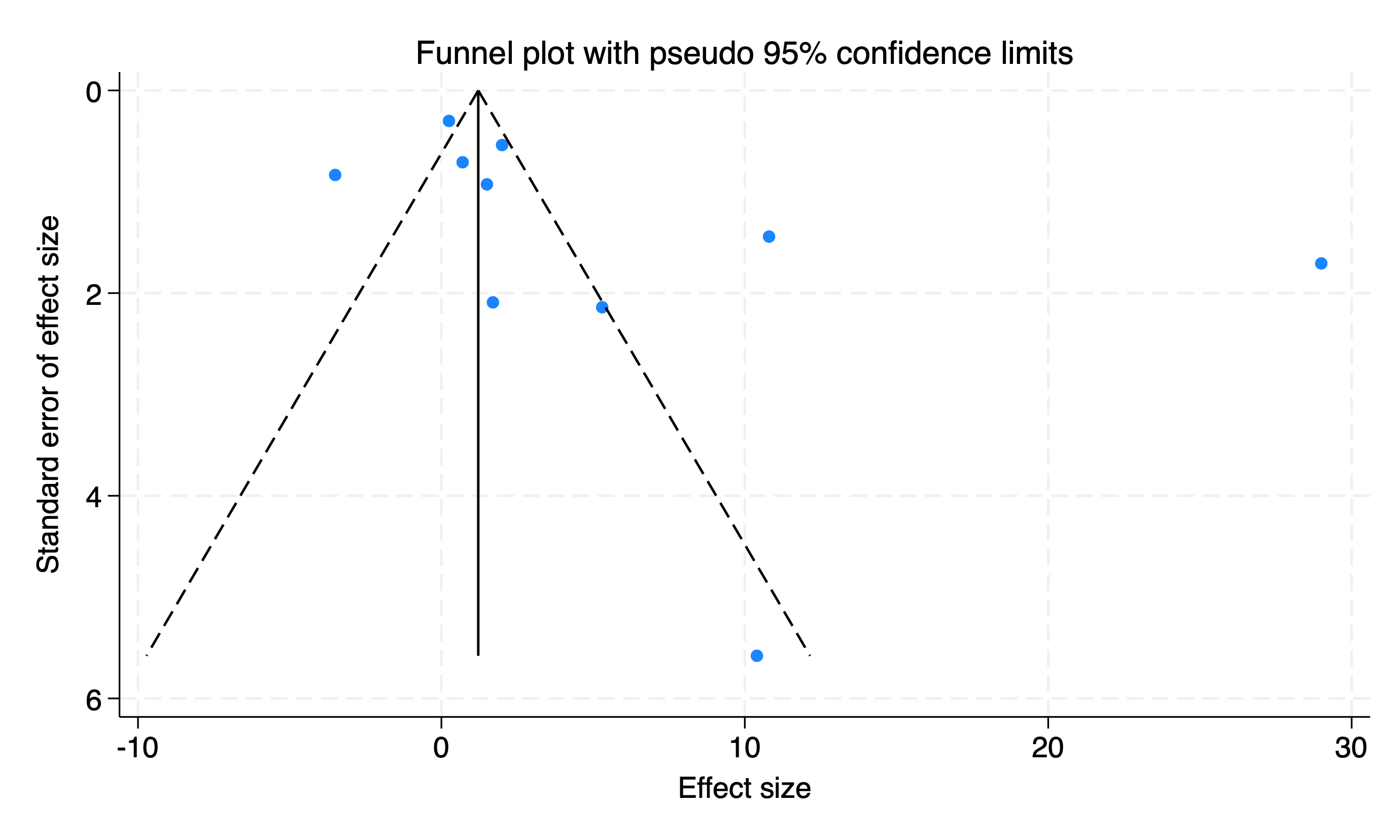

Supplement: SUPPLEMENTARY FIGURE 2 — Funnel plot for impact of sarcopenia on length of hospital stay. [file Image_2.TIF]

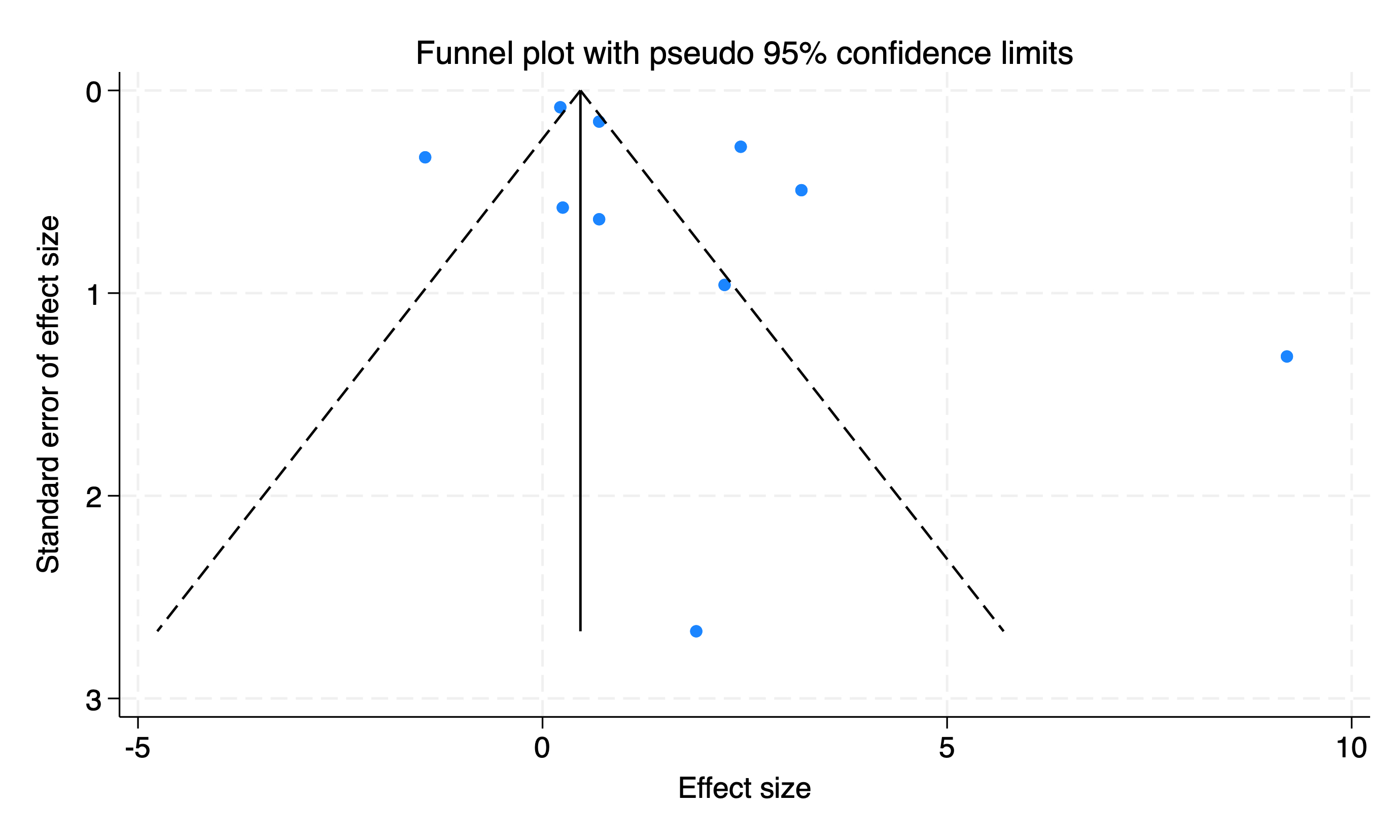

Supplement: SUPPLEMENTARY FIGURE 3 — Funnel plot for impact of sarcopenia on length of ICU stay. [file Image_3.TIF]
