# Supplementary material for: Impact of sarcopenia and frailty on outcomes of patients with sepsis or septic shock: a systematic review and meta-analysis
Source: Front Nutr. 2025 Oct 13;12:1679632. doi: 10.3389/fnut.2025.1679632 (PMC12554568; doi:10.3389/fnut.2025.1679632)
Supplement: Supplementary file 4 [file Data_Sheet_1.PDF]

Supplementary Appendix:

Search Strategy:

PubMed:

("Sepsis"[Mesh] OR "Septic Shock"[Mesh] OR sepsis[Title/Abstract] OR "septic shock"[Title/Abstract] OR septicemia[Title/Abstract] OR "bloodstream infection"[Title/Abstract] OR "bacteremia"[Mesh] OR bacteremia[Title/Abstract] OR "systemic inflammatory response syndrome"[Mesh] OR "systemic inflammatory response syndrome"[Title/Abstract]) AND ("Sarcopenia"[Mesh] OR sarcopenia[Title/Abstract] OR "muscle wasting"[Title/Abstract] OR "skeletal muscle mass"[Title/Abstract] OR "low muscle mass"[Title/Abstract] OR "muscle atrophy"[Mesh] OR "frailty"[Mesh] OR frailty[Title/Abstract] OR "frail"[Title/Abstract] OR "frail elderly"[Mesh] OR "frail elderly"[Title/Abstract] OR "vulnerable elderly"[Mesh] OR "vulnerable elderly"[Title/Abstract] OR "clinical frailty scale"[Title/Abstract] OR "frailty index"[Title/Abstract] OR "frailty phenotype"[Title/Abstract]) AND ("Mortality"[Mesh] OR mortality[Title/Abstract] OR "death"[Title/Abstract] OR "length of stay"[Title/Abstract] OR "Length of Stay"[Mesh] OR "hospital stay"[Title/Abstract] OR "Hospitalization"[Mesh] OR "intensive care units"[Mesh] OR "ICU stay"[Title/Abstract] OR "length of intensive care"[Title/Abstract] OR "length of ICU stay"[Title/Abstract] OR "mechanical ventilation"[Mesh] OR "mechanical ventilation"[Title/Abstract] OR "ventilator"[Title/Abstract] OR "ventilator days"[Title/Abstract] OR "duration of ventilation"[Title/Abstract] OR "mechanical respiratory assistance"[Title/Abstract])

EMBASE:

('sepsis'/exp OR sepsis:ti,ab OR 'septic shock'/exp OR 'septic shock':ti,ab OR septicemia:ti,ab OR 'bloodstream infection':ti,ab OR 'bacteremia'/exp OR bacteremia:ti,ab OR 'systemic inflammatory response syndrome'/exp OR 'systemic inflammatory response syndrome':ti,ab) AND ('sarcopenia'/exp OR sarcopenia:ti,ab OR 'muscle wasting':ti,ab OR 'skeletal muscle mass':ti,ab OR 'low muscle mass':ti,ab OR 'muscle atrophy'/exp OR 'frailty'/exp OR frailty:ti,ab OR frail:ti,ab OR 'frail elderly'/exp OR 'frail elderly':ti,ab OR 'vulnerable elderly'/exp OR 'vulnerable elderly':ti,ab OR 'clinical frailty scale':ti,ab OR 'frailty index':ti,ab OR 'frailty phenotype':ti,ab) AND ('mortality'/exp OR mortality:ti,ab OR death:ti,ab OR 'length of stay':ti,ab OR 'hospitalization'/exp OR 'hospital stay':ti,ab OR 'intensive care unit'/exp OR 'ICU stay':ti,ab OR 'length of ICU stay':ti,ab OR 'mechanical ventilation'/exp OR 'mechanical ventilation':ti,ab OR ventilator:ti,ab OR 'ventilator days':ti,ab OR 'duration of ventilation':ti,ab OR 'mechanical respiratory assistance':ti,ab)

Scopus:

TITLE-ABS-KEY(“sepsis” OR “septic shock” OR “septicemia” OR “bloodstream infection” OR “bacteremia” OR “systemic inflammatory response syndrome”) AND TITLE-ABS-KEY(“sarcopenia” OR “muscle wasting” OR “skeletal muscle mass” OR “low muscle mass” OR “muscle atrophy” OR “frailty” OR “frail” OR “frail elderly” OR “vulnerable elderly” OR “clinical frailty scale” OR “frailty index” OR “frailty phenotype”) AND TITLE-ABS-KEY(“mortality” OR “death” OR “length of stay” OR “hospital stay” OR “hospitalization” OR “ICU stay” OR “length of ICU stay” OR “intensive care unit” OR “mechanical ventilation” OR “ventilator” OR “ventilator days” OR “duration of ventilation” OR “mechanical respiratory assistance”)

CINAHL:

MH “Sepsis” OR MH “Septic Shock” OR TI sepsis OR AB sepsis OR TI “septic shock” OR AB “septic shock” OR TI septicemia OR AB septicemia OR TI “bloodstream infection” OR AB “bloodstream infection” OR MH “Bacteremia” OR TI bacteremia OR AB bacteremia OR MH “Systemic Inflammatory Response Syndrome” OR TI “systemic inflammatory response syndrome” OR AB “systemic inflammatory response syndrome”) AND (MH “Sarcopenia” OR TI sarcopenia OR AB sarcopenia OR TI “muscle wasting” OR AB “muscle wasting” OR TI “skeletal muscle mass” OR AB “skeletal muscle mass” OR TI “low muscle mass” OR AB “low muscle mass” OR MH “Muscle Atrophy” OR TI “muscle atrophy” OR AB “muscle atrophy” OR MH “Frailty” OR TI frailty OR AB frailty OR TI frail OR AB frail OR MH “Frail Elderly” OR TI “frail elderly” OR AB “frail elderly” OR MH “Vulnerable Elderly” OR TI “vulnerable elderly” OR AB “vulnerable elderly” OR TI “Clinical Frailty Scale” OR AB “Clinical Frailty Scale” OR TI “frailty index” OR AB “frailty index” OR TI “frailty phenotype” OR AB “frailty phenotype”) AND (MH “Mortality” OR TI mortality OR AB mortality OR TI death OR AB death OR TI “length of stay” OR AB “length of stay” OR MH “Length of Stay” OR TI “hospital stay” OR AB “hospital stay” OR MH “Hospitalization” OR TI hospitalization OR AB hospitalization OR MH “Intensive Care Units” OR TI “ICU stay” OR AB “ICU stay” OR TI “length of ICU stay” OR AB “length of ICU stay” OR MH “Mechanical Ventilation” OR TI “mechanical ventilation” OR AB “mechanical ventilation” OR TI ventilator OR AB ventilator OR TI “ventilator days” OR AB “ventilator days” OR TI “duration of ventilation” OR AB “duration of ventilation” OR TI “mechanical respiratory assistance” OR AB “mechanical respiratory assistance”)

Web of Science:

TS=(“sepsis” OR “septic shock” OR “septicemia” OR “bloodstream infection” OR “bacteremia” OR “systemic inflammatory response syndrome”) AND TS=(“sarcopenia” OR “muscle wasting” OR “skeletal muscle mass” OR “low muscle mass” OR “muscle atrophy” OR “frailty” OR “frail” OR “frail elderly” OR “vulnerable elderly” OR “clinical frailty scale” OR “frailty index” OR “frailty phenotype”) AND TS=(“mortality” OR “death” OR “length of stay” OR “hospital stay” OR “hospitalization” OR “ICU stay” OR “length of ICU stay” OR “intensive care unit” OR “mechanical ventilation” OR “ventilator” OR “ventilator days” OR “duration of ventilation” OR “mechanical respiratory assistance”)

Cochrane library:

(MeSH descriptor: [Sepsis] explode all trees OR sepsis:ti,ab,kw OR “septic shock”:ti,ab,kw OR septicemia:ti,ab,kw OR “bloodstream infection”:ti,ab,kw OR (MeSH descriptor: [Bacteremia] explode all trees) OR bacteremia:ti,ab,kw OR (MeSH descriptor: [Systemic Inflammatory Response Syndrome] explode all trees) OR “systemic inflammatory response syndrome”:ti,ab,kw) AND (MeSH descriptor: [Sarcopenia] explode all trees OR sarcopenia:ti,ab,kw OR “muscle wasting”:ti,ab,kw OR “skeletal muscle mass”:ti,ab,kw OR “low muscle mass”:ti,ab,kw OR (MeSH descriptor: [Muscle Atrophy] explode all trees) OR “muscle atrophy”:ti,ab,kw OR (MeSH descriptor: [Frailty] explode all trees) OR frailty:ti,ab,kw OR frail:ti,ab,kw OR (MeSH descriptor: [Frail Elderly] explode all trees) OR “frail elderly”:ti,ab,kw OR (MeSH descriptor: [Vulnerable Populations] explode all trees) OR “vulnerable elderly”:ti,ab,kw OR “clinical frailty scale”:ti,ab,kw OR “frailty index”:ti,ab,kw OR “frailty phenotype”:ti,ab,kw) AND ((MeSH descriptor: [Mortality] explode all trees) OR mortality:ti,ab,kw OR death:ti,ab,kw OR “length of stay”:ti,ab,kw OR (MeSH descriptor: [Length of Stay] explode all trees) OR hospitalization:ti,ab,kw OR (MeSH descriptor: [Hospitalization] explode all trees) OR “hospital stay”:ti,ab,kw OR (MeSH descriptor:

[Intensive Care Units] explode all trees) OR “ICU stay”:ti,ab,kw OR “length of ICU stay”:ti,ab,kw OR (MeSH descriptor: [Respiration, Artificial] explode all trees) OR “mechanical ventilation”:ti,ab,kw OR ventilator:ti,ab,kw OR “ventilator days”:ti,ab,kw OR “duration of ventilation”:ti,ab,kw)
